# Association of Serum 25-Hydroxyvitamin D Level With Metabolic Phenotypes of Obesity in Children and Adolescents: The CASPIAN-V Study

**DOI:** 10.3389/fendo.2020.00310

**Published:** 2020-06-16

**Authors:** Haleh Esmaili, Ramin Heshmat, Hanieh-Sadat Ejtahed, Hadith Rastad, Mohammad Esmaeil Motlagh, Hamid Asayesh, Marzieh Jafarnejad, Ehsan Seif, Mostafa Qorbani, Roya Kelishadi

**Affiliations:** ^1^Pediatrics Department, Child Growth and Development Research Center, Research Institute for Primordial Prevention of Non-Communicable Disease, Isfahan University of Medical Sciences, Isfahan, Iran; ^2^Social Determinants of Health Research Center, Health Research Institute, Babol University of Medical Sciences, Babol, Iran; ^3^Chronic Diseases Research Center, Endocrinology and Metabolism Population Sciences Institute, Tehran University of Medical Sciences, Tehran, Iran; ^4^Endocrinology and Metabolism Research Center, Endocrinology and Metabolism Clinical Sciences Institute, Tehran University of Medical Sciences, Tehran, Iran; ^5^Social Determinants of Health Research Center, Alborz University of Medical Sciences, Karaj, Iran; ^6^Pediatrics Department, Ahvaz Jundishapur University of Medical Sciences, Ahvaz, Iran; ^7^Department of Medical Emergencies, Qom University of Medical Sciences, Qom, Iran; ^8^Student Research Committee, Alborz University of Medical Sciences, Karaj, Iran; ^9^Non-Communicable Diseases Research Center, Alborz University of Medical Sciences, Karaj, Iran; ^10^Department of Epidemiology, Chronic Diseases Research Center, Endocrinology and Metabolism Population Sciences Institute, Tehran University of Medical Sciences, Tehran, Iran

**Keywords:** obesity, 25-hydroxyvitamin D, children and adolescents, metabolic syndrome, metabolically healthy obese

## Abstract

**Background and Objective:** Different metabolic phenotypes of obesity are related to cardiometabolic risk factors in children and adolescents. Vitamin D, as one important factor, could be related to different subgroups of metabolic obesity and might affect metabolic disorders. The purpose of this study was to evaluate the relationship between serum 25-hydroxyvitamin D concentration and subsets of metabolic phenotypes of obesity in children and adolescents.

**Methods:** This nationwide cross-sectional study was conducted in the framework of the fifth survey of a national surveillance program, the CASPIAN study. Overall, 2,594 students aged 7–18 years were assessed for 25-hydroxyvitamin D status. Metabolic syndrome (MetS) was defined according to the ATP III criteria modified for the pediatric age group. Participants were classified into four metabolic phenotypes of obesity according to categories of the BMI and metabolic status: “metabolically healthy obese” (MHO), “metabolically non-healthy non-obese” (MNHNO), “metabolically non-healthy obese” (MNHO), and “metabolically healthy non-obese” (MHNO). Multinomial logistic regression analysis was performed for evaluating the association of 25-hydroxyvitamin D status with different metabolic phenotypes of obesity.

**Results:** In this study, 85.2% of participants were classified as MHNO, 11.0 % as MHO, 2.5% as MNHNO, and 1.3% as MNHO. The frequency of hypovitaminosis D was more prevalent in MNHO (85.3%) than in other phenotypes (MHNO: 70%; MHO: 76.5%; MNHNO: 78.1%, respectively; *p* < 0.05). In the multivariate model, hypovitaminosis D significantly increased the odds of being MHO (OR: 1.46; 95% CI: 1.07–1.77) and MNHO (OR: 2.89; 1.05–8.31) compared to the healthy group. Likewise, in multivariate model, per each unit (ng/mL) increment in 25-hydroxyvitamin D concentration, the odds of MNHNO and MNHO decreased significantly by 7% (OR: 0.93; 0.91–0.96) and 6% (OR: 0.94; 0.91–0.98) respectively.

**Conclusion:** Our results support the hypothesis that 25-hydroxyvitamin D concentration is associated with metabolic obesity phenotypes. Longitudinal studies are necessary to assess the clinical impacts of this finding.

## Introduction

Obesity, especially among children and adolescents, is a major health challenge in developed and developing countries (1–3). It is also an important risk factor for the development of metabolic diseases, including diabetes mellitus (DM) and hypertension, as well as an increase in serum total cholesterol (TC) and triglycerides (TG) (4). Despite this fact, some studies recognized non-obese individuals with metabolic abnormality—for instance, low high-density lipoprotein-cholesterol (HDL-C), high TG and TC (5, 6). These subjects were known as metabolically non-healthy non-obese (MNHNO). On the other hand, some evidence exists on obese patients without any type of metabolic disorders, they are considered as metabolically healthy obese (MHO) group (7–9). It is reported that the prevalence of metabolic syndrome (MetS) is 29.2% in obese children (10).

Vitamin D plays numerous extra-skeletal roles and functions. This fat-soluble vitamin has an inverse association with insulin resistance, obesity, and MetS. It is shown that individuals in the MHO group had a higher concentration of serum 25-hydroxyvitamin D than the metabolic non-healthy obese (MNHO) group (11, 12). However, data regarding serum vitamin D level and metabolic phenotypes of obesity among children and adolescents are limited. The current findings are controversial, and some studies did not show any significant association between vitamin D and various metabolic phenotypes of obesity (13, 14). Different variables as age, gender, and habitat might influence the reports of such association in adolescents; therefore, more studies are needed in this field (15). This study aimed to investigate the prevalence of obesity subsets and their relationships with serum 25-hydroxyvitamin D among Iranian children and adolescents.

## Materials and Methods

### Study Population

This cross-sectional study was conducted as part of the fifth phase of a national school-based survey entitled “Childhood and Adolescence Surveillance and Prevention of Adult Non-communicable Disease” (CASPIAN-V study), which has been described in detail previously (16). The protocol of this study was mainly based on the World Health Organization-Global School Student Health Survey. Briefly, 14,400 students aged 7–18 years from primary and secondary schools in urban and rural areas of 30 provinces throughout the Iran were selected using multistage, stratified cluster sampling method in 2015 (March to May). The number of selected boys and girls was the same in each province, and the sample size in urban and rural areas was proportional to the number of students in different areas. In the next step, 4,200 students were randomly selected for biochemical tests, from which 2,594 samples have been assessed for 25-hydroxyvitamin D status. This study was conducted according to the guidelines of the Declaration of Helsinki. After explaining the study objectives and methods, written informed consent and verbal assent were obtained from all parents and students, respectively. The study protocol was reviewed and approved by the Research and Ethics Council of the National Institute for Medical Research Development (NIMAD) (project number: 194049).

### Questionnaires

The demographic questionnaire was completed for the pediatric age group. All students were asked about the consumption of vitamin D supplements. The screen time (ST) was assessed as the average number of hours per day spent watching television or videos, using their personal computer, or playing electronic games by a validated questionnaire (17). According to the international ST recommendations, ST was classified into two groups (low: <2 h per day and high: equal or more than 2 h per day (18).

Physical activity (PA) of students in the prior week was assessed using a validated questionnaire. Participants reported the frequency of their leisure-time physical activity outside of school, which caused heavy sweating or large increases in breathing or heartrate and lasted at least 30 min. PA was categorized into two groups (low: 0–3 days per week and high: 4–7 days per week) (17). For calculating the family's socioeconomic status (SES), questions about the parental occupational status, parental level of education, school type (public/private), having a private car, and possessing a personal computer were included in the questionnaire. SES was classified into three levels (low/moderate/high).

### Anthropometric Measurements and Laboratory Tests

Trained healthcare providers conducted anthropometric measurements. Height was measured in a standing position without shoes to the nearest 0.5 cm. Weight was measured on a digital scale (SECA, Germany) placed on flat ground with minimal clothing and without shoes to the nearest 0.1 kg. Body mass index (BMI) was calculated as weight (kg) divided by square of height (m^2^). Waist circumference (WC) was measured at the midpoint between the lower margin of the rib cage and top of the iliac crest at the end of normal expiration using a non-elastic tape with 0.1 cm accuracy (19). Blood pressure (BP) was measured twice with a 5-min interval on the right arm in a sitting position, using a standardized mercury sphygmomanometer. Systolic blood pressure (SBP) and diastolic blood pressure (DBP) were considered as the first and fifth Korotkoff sounds, respectively. The average of two measurements was considered as the BP.

Fasting blood samples were collected in the morning after 12–14 h of overnight fasting from all students. Biochemical variables, including fasting blood glucose (FBG), TC, HDL-C, and TG were measured enzymatically by Hitachi auto-analyzer (Tokyo, Japan) (20). The serum concentration of 25-hydroxyvitamin D was measured by a direct competitive immunoassay chemiluminescent method using LIASON 25-hydroxyvitamin D assay TOTAL (DiaSorin, Inc.) with a coefficient of variation (CV) of 9.8%.

### Diagnostic Criteria

The World Health Organization (WHO) growth curves were used for BMI classification of pediatrics. General obesity in students was defined as age and sex-specific BMI more than 95th percentile, overweight was defined as age and sex-specific BMI between the 85th to 95th percentiles, and normal weight was defined as age and sex-specific BMI between the 5th to 85th percentiles. According to the Adult Treatment Panel III (ATP III) criteria with modification for the child age group, MetS was defined as the presence of three or more of the following components: (1) serum TG concentration of 150 mg/dl or greater; (2) serum HDL-C concentration of 40 mg/dL or less; (3) serum FBG level of 100 mg/dl or greater; (4) abdominal obesity defined as waist to height ratio > 0.5; and (5) either SBP or DBP greater than the 90th percentile for age, sex, and height (21). Students were classified into four metabolic phenotypes of obesity based on their BMI and metabolic status: (1) metabolically healthy non-obese (MHNO): normal weight pediatrics who is characterized by the absence of MetS; (2) MHO: obese pediatrics who is characterized by the absence of MetS; (3) MNHO: obese children with metabolic syndrome; (4) MNHNO: normal-weight children with MetS (22). A serum 25-hydroxyvitamin D level of <10 ng/mL was defined as vitamin D deficiency, between 10 and 30 ng/mL as vitamin D insufficiency, and more than 30 ng/mL as vitamin D sufficiency. For purposes of the analysis, we defined hypovitaminosis D as 25-hydroxyvitamin D <30 ng/mL, because this is a recognized cutoff for healthy vitamin D concentrations (23–25).

### Statistical Analysis

All variables were checked for normality and are presented as median (inter-quartile range), mean (standard deviation), or number (percentage). Demographic characteristics and metabolic phenotypes of obesity were compared according to gender, using independent sample *t*-test, Mann-Whitney test, and Chi-square test. The prevalence of hypovitaminosis D was compared among different metabolic phenotypes of obesity by the Chi-square test. Association of hypovitaminosis D with different metabolic phenotypes of obesity was examined using nominal logistic regression analysis adjusted for potential confounders, including age, sex, living area, screen time, and SES, physical activity, and vitamin D supplementation. Results are presented as odds ratios (OR) with a 95% confidence interval (CI). Multinomial logistic regression analysis was performed for evaluating the association of 25-hydroxyvitamin D concentration with different metabolic phenotypes of obesity. Three models were defined: Model I represented the crude association without any adjustment; Model II adjusted for age, sex, and living place; and Model III additionally adjusted for ST, PA, SES, and vitamin D supplementation. Data analysis was performed using STATA version 11.0 (STATA Statistical Software: Release 11. StataCorp LP. Package, College Station, TX, USA). *P*-value of less than 0.05 was considered as statistically significant.

## Results

**Figure 1:**
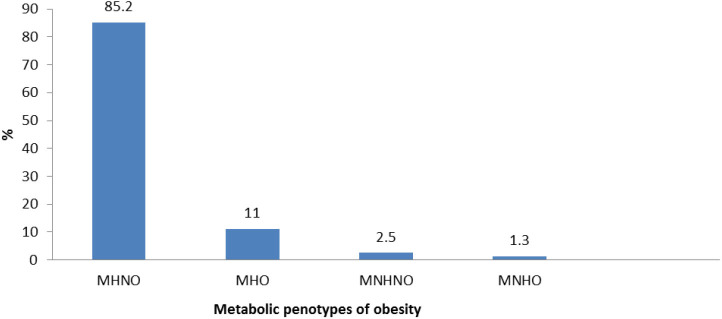
Prevalence of different metabolic phenotypes of obesity in Iranian children and adolescents: the CASPIAN-V study (*n* = 2,594). MHNO, metabolically healthy non obese; MHO, metabolically healthy obese; MNHNO, metabolically non healthy non obese; MNHO, metabolically non healthy obese.

A total of 2,596 children and adolescents aged 7–18 years were included in this study. Overall, the mean (SD) age was 12.2 (3.1) years; 44.9% (*n* = 1,166) were girls, and 71.3% (*n* = 1,850) were urban residents. The median (interquartile range) of 25-hydroxyvitamin D was 26.49 (10.0); 71.1% (817) of subjects had hypovitaminosis D. MHNO was identified in 2,211 (85.2%), MHO in 285 (11.0%), MNHNO in 64 (2.5%), and MNHO in 34 (1.3 %) subjects (Figure 1).

Table 1 presents the demographic characteristics and metabolic phenotypes of obesity according to gender. There was a significant difference between boys and girls in terms of obesity phenotypes (*p* = 0.009); a higher percentage of boys and girls had MHNO (83.5 and 87.4% respectively), and the lowest percentage was MNHO (1.7% in boys and 0.9% in girls). Serum 25-hydroxyvitamin D concentration and prevalence of hypovitaminosis D were not statistically different between boys and girls (both *p*-values > 0.05).

**Table 1:** Demographic characteristics and metabolic phenotypes of obesity according to gender: the CASPIAN-V study.

**Variable**	**Girls *N* = 1,166**	**Boys *N*= 1,430**	***p*-value**
**Age (years)^a^**	12.0 (3.1)	12.3 (3.0)	0.006
**Living area^b^**			
Urban	828 (71.0)	1022 (71.5)	0.79
Rural	338 (29.0)	408 (28.5)	
**Obesity phenotype^b^**			
MHNO	1,019 (87.4)	1,192 (83.5)	0.009
MHO	106 (9.1)	179 (12.5)	
MNHNO	31 (2.7)	33 (2.3)	
MNHO	10 (0.9)	24 (1.7)	
**25-hydroxyvitamin D concentration (ng/mL)^c^**	25.62 (10.51)	26.83 (9.00)	0.33
**Vitamin D status^b^**			
Hypovitaminosis	817 (70.1)	1,029 (72.0)	0.29
Normal	349 (29.9)	401 (28.0)	
**Vitamin D supplement use^b^**	42 (3.6)	44 (3.1)	0.45
**Physical activity**			
Low	699 (60.3)	777 (54.6)	0.004
High	461 (39.7)	647 (45.4)	
**Screen time^b^**			
Low	985 (86.40)	1,179 (84.8)	0.24
High	155 (13.6)	212 (15.2)	
**SES^b^**			
Low	403 (36.6)	405 (29.4)	<0.001
Moderate	331 (30.1)	496 (36.0)	
High	366 (33.3)	477 (34.6)	

*Vitamin D insufficiency: 25-hydroxyvitamin D <30 ng/mL*.

*MHNO, metabolically healthy non obese; MHO, metabolically healthy obese; MNHNO, metabolically non healthy non obese; MNHO, metabolically non healthy obese; SES, socioeconomic state*.

a *are presented as mean (SD) and compared using t-test*.

b *are presented as number (percentage) and compared using Chi-square test*.

c *are presented as median (inter-quartile range) and compared using Mann–Whitney test*.

**Figure 2:**
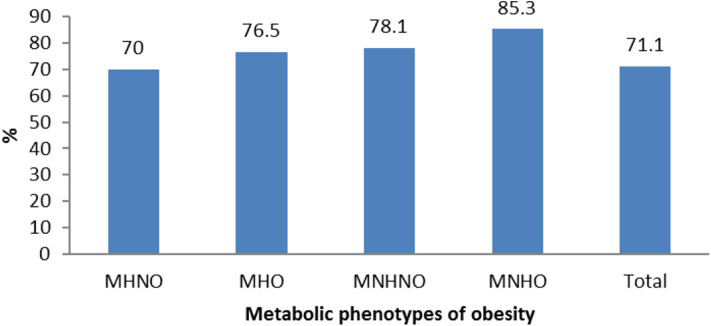
Prevalence of hypovitaminosis D according to different metabolic phenotypes of obesity in Iranian children and adolescents: the CASPIAN-V study. MHNO, metabolically healthy non obese; MHO, metabolically healthy obese; MNHNO, metabolically non healthy non obese; MNHO, metabolically non healthy obese.

As shown in Figure 2, the prevalence of vitamin D deficiency had a significant difference among different metabolic phenotypes of obesity (*p* < 0.001); it was more prevalent in MNHO (85.3%) than in other phenotypes.

Based on the crude model of nominal logistic regression, serum 25-hydroxyvitamin D <30 ng/mL significantly increased odds of being MHO rather than being MHNO by 1.39 times (95% CI: 1.05–1.86); this association was not changed after adjustment for other variables. Besides, according to the adjusted model for ST, PA, SES, and vitamin D supplementation, serum 25-hydroxyvitamin D <30 ng/mL significantly increased odds of being MNHO rather than being MHNO by 2.89 times (95 % CI: 1.05–8.31). There was no significant association between hypovitaminosis D and other metabolic phenotypes of obesity (Table 2).

**Table 2:** Association of hypovitaminosis D with different metabolic phenotypes of obesity in nominal logistic regression: the CASPIAN-V study.

	**Metabolic phenotypes of obesity**			
	**MHNO**	**MHO**	**MNHNO**	**MNHO**
**Hypovitaminosis D**				
Model I	Reference	1.39 (1.05–1.86)^*^	1.53 (0.84–2.79)	2.48 (0.95–6.45)
Model II	Reference	1.37(1.30–1.83)^*^	1.51 (0.83–2.75)	2.44 (0.94–6.35)
Model III	Reference	1.46(1.07–1.77)^*^	1.29 (0.70–2.39)	2.89 (1.05–8.31)^*^

*Hypovitaminosis D: 25-hydroxyvitamin D <30 ng/mL*.

*The model I: crude association; Model II: adjust for age, gender, living place; Model III: adjust for ST, PA, SES, and vitamin D supplementation. MHNO, metabolically healthy non-obese; MHO, metabolically healthy obese; MNHNO, metabolically non healthy non-obese; MNHO, metabolically non-healthy obese*.

*Values are reported as OR (95% CI)*.

* *Statistically significant (p < 0.05)*.

As presented in Table 3, each unit increase in serum 25-hydroxyvitamin D concentration significantly decrease the odds of being MHO, MNHO, and MNHNO by 2, 4, and 8%, respectively. Similar associations were also observed in the adjusted model for age, gender, and living area, but in the multivariate model (Model III), the association between 25-hydroxyvitamin D concentration and MHO did not reach statistical significance.

**Table 3:** Association of vitamin D concentration with different metabolic phenotypes of obesity in nominal logistic regression: the CASPIAN-V study.

	**Metabolic phenotypes of obesity**			
	**MHNO**	**MHO**	**MNHNO**	**MNHO**
**Vitamin D concentration (ng/mL)**				
Model I	Reference	0.98 (0.97–0.99)^*^	0.92 (0.90–0.95)^*^	0.96 (0.92–0.99)^*^
Model II	Reference	0.97 (0.97–0.99)^*^	0.93 (0.90–0.95)^*^	0.95 (0.92–0.98)^*^
Model III	Reference	0.99 (0.98–1.01)	0.93 (0.91–0.96)^*^	0.94 (0.91–0.98)^*^

*Model I: crude association; Model II: adjust for age, sex, living place; Model III: adjust for ST, PA, SES, vitamin D supplementation*.

*MHNO, metabolically healthy non obese; MHO, metabolically healthy obese; MNHNO, metabolically non healthy non obese; MNHO, metabolically non healthy obese*.

*Values are reported as OR (95% CI)*.

* *Statistically significant (p < 0.05)*.

*OR calculated per each unit (ng/mL) increment in vitamin D concentration*.

## Discussion

To the best of our knowledge, the current study is the first nationwide study of its kind, not only in Middle Eastern countries, but also in developing countries. The present study evaluated the relationship between vitamin D status and various metabolic phenotypes of obesity. Our data indicated that vitamin D status and metabolic health have significant interaction in children and adolescents. The prevalence of hypovitaminosis D was markedly lower in the MNHO group, compared to those in the MHNO group. Moreover, the prevalence of hypovitaminosis D was significantly higher among MNHO, followed by MNHNO and MHO groups. In adjusted logistic regression models, hypovitaminosis D significantly increased the odds of MNHO, MNHNO, and MHO, respectively. These findings were in line with the previous studies (26–29). Hypovitaminosis D showed a slightly stronger association with MNHO than other phenotypes of obesity. This finding was in contrast to Minambres et al. study, which showed that association of vitamin D and MetS is independent of the degree of obesity (30). The main hypotheses that can justify the association of hypovitaminosis D with obesity and MetS are less exposure to sunlight, an inadequate intake of vitamin D, and decreased bioavailability of vitamin D (31).

There is some controversy regarding the relationship between 25-hydroxyvitamin D concentrations and different metabolic phenotypes of obesity. Some studies showed that vitamin D deficiency was related to a higher risk of diabetes, cardiovascular diseases, and cardiometabolic risk factors among adolescents by elevated the prevalence of adiposity (32–35). Inversely, Hong et al. did not find any significant difference in vitamin D levels between MHO and MNHO groups (8). The same result was found among 8-18-year African-Caribbean-American children and adolescents, showing no significant relationship between vitamin D and phenotypes of metabolic obesity (15). Our findings are in line with a previous study in Iranian adults that showed significant differences between metabolic phenotypes of obesity and 25-hydroxyvitamin D concentration. However, contrary to that study, we found that the MHO group had significantly higher 25-hydroxyvitamin D concentration than other groups. In our study, the latter was observed in the MHNO group. Besides, the study in Iranian adults found a significant relationship between vitamin D status and cardiometabolic risk factors (36).

Nevertheless, it should be noted that unhealthy metabolic profile had a bidirectional association to decrease in vitamin D and obesity related to at least five risk factors of MetS (36, 37). Our study is in agreement with the study of the Third National Health and Nutrition Examination Survey (NHANES III) that found a significant relationship between vitamin D and metabolic health. It also demonstrated that vitamin D was inversely associated with cardiometabolic mortality in MNHO young adults, as well as with a decrease in the prevalence of adiposity in children (38, 39). Although hypovitaminosis D is observed in obese individuals, it is shown that vitamin D supplementation did not have an association with weight reduction (40). Our findings are in agreement with this study showing that an increase in 25-hydroxyvitamin D increased the odds of being MNHNO. Moreover, no or weak association was found between serum 25-hydroxyvitamin D and cardiometabolic risk factors, and the relationship between 25-hydroxyvitamin D and TC was observed only in the obese groups (13, 14, 41, 42). Consistently, our study showed hypovitaminosis D increased the odds of metabolic health obesity rather than other groups even after adjusting for gender, living area, physical activity, ST, and SES. Inconsistent with some other studies, a Korean study showed a negative association between vitamin D concentration and elevated fasting blood sugar (13). One of the reasons for this debate and controversy between studies may be because of differences in various definitions used for metabolic phenotypes of obesity. Another reason can be the difference in ethnicity, living region, and race that might affect 25-hydroxyvitamin D concentrations; moreover, it can be affected by body fat distribution and definition of obesity and metabolic obesity subgroups.

Furthermore, the current study addressed the prevalence of different metabolic phenotypes of obesity in Iranian children and adolescents. The prevalence of MHNO was higher than other groups, followed by MHO, MNHNO, and MNHO, respectively. This finding was in line with previous studies in Iranian children and adolescents (43, 44).

The prevalence of various metabolic phenotypes of obesity was different according to age, race, living area, occupation, and the definition of risk factors (45–47). Therefore, according to the previous studies, the prevalence of MHO varied from 10 to 40% among adults (8, 48, 49). Besides, among obese boys and girls, the prevalence of MHO ranged from 3% in African-Americans to about 47.7% in Koreans (8, 49–51). It is also showed that among 8–18 year-old pupils, the frequency of MHO was 20–53% (15, 52, 53). We suggest that as in the current study, the individuals' characteristics and ethnicity were different than other studies; the prevalence of MHO was lower than this range. According to the above-mentioned studies, the Asian population showed a higher number of MHO than Western people; however, our findings in Iranian children and adolescents, as an Asian country, are not consistent with this issue.

The large sample size is one of the main strengths of this study. To the best of our knowledge, the current study is the first nationwide study in this field, not only in the Middle East, but also in developing countries. Thus, future studies are needed to have a better understanding of this interaction in various ethnic populations.

The main limitation of our study is the cross-sectional nature of the data. The other limitation is that the groups of obesity phenotypes did not have homogeny, as the MNHNO and MNHO groups had a small sample size. Moreover, we could not examine the pubertal status of participants in our school-based study, and puberty influences the variables assessed in our study. Measurement of 25-hydroxyvitamin D using immunoassay chemiluminescent method is another limitation of this study.

This is an association study that cannot provide causation. Randomized trials and longitudinal studies would be required to assess if treatment/supplementation with vitamin D could improve the metabolic profile of various obesity phenotypes.

In conclusion, our study examined the four subsets of metabolic obesity and 25-hydroxyvitamin D concentration as an associated factor on metabolic phenotypes of obesity in Iranian pediatric population. According to our results, we found hypovitaminosis D is related to an increase in odds of MHO, and an increase in 25-hydroxyvitamin D concentration declines the odds of MHO, MNHNO, and MNHO, significantly. The study on different metabolic phenotypes of obesity, especially in metabolically abnormal groups of obesity in children and adolescents, can be useful to evaluate the difference of metabolic obesity subgroups and the related factors. It can be of help for efficient prevention and treatment guidelines, as well as and strategies and modify lifestyle.

## Data Availability Statement

All datasets generated for this study are included in the article/supplementary material.

## Ethics Statement

The studies involving human participants were reviewed and approved by Research and Ethics Council of Isfahan University of Medical Sciences (project number: 194049). Written informed consent to participate in this study was provided by the participants' legal guardian/next of kin.

## Author Contributions

RK, MQ, RH, and MM: design. RK, MQ, RH, MM, ES, MJ, and HA: study conduct. MQ: statistical analysis. HE, H-SE, and HR: paper drafting. All authors have contributed to revising the manuscript and confirmed the final draft for submission and accept the responsibility of the manuscript content.

## Conflict of Interest

The authors declare that the research was conducted in the absence of any commercial or financial relationships that could be construed as a potential conflict of interest. The handling editor declared a past co-authorship with the authors MQ, MM, RH, and RK.

## References

[1] Farello G Antenucci A Ceci F Ambrosi M Verrotti A Anthropometric and metabolic parameters to distinguish metabolically healthy obese children from children with metabolic syndrome. Group. (2017) 16:18 10.1186/s12872-018-0874-5

[2] Djalalinia S Qorbani M Peykari N Kelishadi R. Health impacts of obesity. Pakistan J Med Sci. (2015) 31:239. 10.12669/pjms.311.7033 25878654 PMC4386197

[3] Rahmanian M Kelishadi R Qorbani M Motlagh ME Shafiee G Aminaee T. Dual burden of body weight among Iranian children and adolescents in 2003 and 2010: the CASPIAN-III study. Arch Med Sci. (2014) 10:96. 10.5114/aoms.2014.40735 24701221 PMC3953979

[4] Muñoz-Garach A Cornejo-Pareja I Tinahones FJ. Does metabolically healthy obesity exist? Nutrients. (2016) 8:320. 10.3390/nu8060320 27258304 PMC4924161

[5] Ruderman N Chisholm D Pi-Sunyer X Schneider S. The metabolically obese, normal-weight individual revisited. Diabetes. (1998) 47:699–713. 10.2337/diabetes.47.5.699 9588440

[6] Kelishadi R Motlagh ME Roomizadeh P Abtahi SH Qorbani M Taslimi M First report on path analysis for cardiometabolic components in a nationally representative sample of pediatric population in the Middle East and North Africa (MENA): the CASPIAN-III Study. Ann Nutr Metab. (2013) 62:257–65. 10.1159/000346489 23635794

[7] Blüher S Schwarz P. Metabolically healthy obesity from childhood to adulthood—does weight status alone matter? Metabolism. (2014) 63:1084–92. 10.1016/j.metabol.2014.06.009 25038727

[8] Hong HC Lee J-S Choi HY Yang SJ Yoo HJ Seo JA. Liver enzymes and vitamin D levels in metabolically healthy but obese individuals: Korean National Health and Nutrition Examination Survey. Metabolism. (2013) 62:1305–12. 10.1016/j.metabol.2013.04.002 23643404

[9] Sims EA. Are there persons who are obese, but metabolically healthy? Metab Clin Exp. (2001) 50:1499–504. 10.1053/meta.2001.27213 11735101

[10] Friend A Craig L Turner S. The prevalence of metabolic syndrome in children: a systematic review of the literature. Metab Syndr Relat Disord. (2013) 11:71–80. 10.1089/met.2012.0122 23249214

[11] Minambres I Sánchez-Quesada JL Vinagre I Sánchez-Hernández J Urgell E de Leiva A. Hypovitaminosis D in type 2 diabetes: relation with features of the metabolic syndrome and glycemic control. Endocr Res. (2015) 40:160–5. 10.3109/07435800.2014.982326 25536005

[12] Asadi M Matin N Frootan M Mohamadpour J Qorbani M Tanha FD. Vitamin D improves endometrial thickness in PCOS women who need intrauterine insemination: a randomized double-blind placebo-controlled trial. Arch Gynecol Obstetr. (2014) 289:865–70. 10.1007/s00404-013-3055-x 24158736

[13] Baker CP Kulkarni B Radhakrishna K Charyulu M Gregson J Matsuzaki D. Is the association between vitamin D and cardiovascular disease risk confounded by obesity? PLoS ONE, 10, e0129468. 10.1371/journal.pone.0129468 26079685 PMC4469320

[14] Challa AS Makariou SE Siomou EC. The relation of vitamin D status with metabolic syndrome in childhood and adolescence: an update. J Pediatr Endocrinol Metab. (2015) 28:1235–45. 10.1515/jpem-2014-0485 26053006

[15] Khokhar A Chin V Perez-Colon S Farook T Bansal S Kochummen E. Differences between metabolically healthy vs unhealthy obese children and adolescents. J Natl Med Assoc. (2017) 109:203–10. 10.1016/j.jnma.2017.02.008 28987250

[16] Motlagh ME Ziaodini H Qorbani M Taheri M Aminaei T Goodarzi A. Methodology and early findings of the fifth survey of childhood and adolescence surveillance and prevention of adult noncommunicable disease: The CASPIAN-V study. Int J Prevent Med. (2017) 8:915. 10.4103/2008-7802.198915 28217266 PMC5288959

[17] Kelishadi R Majdzadeh R Motlagh M-E Heshmat R Aminaee T Ardalan G. Development and evaluation of a questionnaire for assessment of determinants of weight Development and Evaluation of a Questionnaire for Assessment of Determinants of Weight disorders among children and adolescents: the caspian-IV Study. Int J Prevent Med. (2012) 3:699. 23112896 PMC3482997

[18] Strong WB Malina RM Blimkie CJ Daniels SR Dishman RK Gutin B. Evidence based physical activity for school-age youth. J Pediatric. (2005) 146:732–7. 10.1016/j.jpeds.2005.01.055 15973308

[19] World Health Organization Physical Status: The Use of and Interpretation of Anthropometry. Report of a WHO Expert Committee (1995). 8594834

[20] McNamara JR Schaefer EJ. Automated enzymatic standardized lipid analyses for plasma and lipoprotein fractions. Clin Chim Acta. (1987) 166:1–8. 10.1016/0009-8981(87)90188-4 3608193

[21] Zimmet P Alberti G Kaufman F Tajima N Silink M Arslanian S. The metabolic syndrome in children and adolescents. Lancet. (2007) 369:2059–61. 10.1016/S0140-6736(07)60958-1 17586288

[22] Damanhoury S Newton A Rashid M Hartling L Byrne J Ball G. Defining metabolically healthy obesity in children: a scoping review. Obesity Rev. (2018) 19:1476–91. 10.1111/obr.12721 30156016

[23] Heaney RP. Functional indices of vitamin D status and ramifications of vitamin D deficiency. Am J Clin Nutrit. (2004) 80:1706S−9S. 10.1093/ajcn/80.6.1706S 15585791

[24] Bischoff-Ferrari HA Giovannucci E Willett WC Dietrich T Dawson-Hughes B. Estimation of optimal serum concentrations of 25-hydroxyvitamin D for multiple health outcomes. Am J Clin Nutr. (2006) 84:18–28. 10.1093/ajcn/84.1.18 16825677

[25] Weng FL Shults J Leonard MB Stallings VA Zemel BS. Risk factors for low serum 25-hydroxyvitamin D concentrations in otherwise healthy children and adolescents. Am J Clin Nutr. (2007) 86:150–8. 10.1093/ajcn/86.1.150 17616775

[26] Taheri E Saedisomeolia A Djalali M Qorbani M Civi MM. The relationship between serum 25-hydroxy vitamin D concentration and obesity in type 2 diabetic patients and healthy subjects. J Diabetes Metab Disord. (2012) 11:16. 10.1186/2251-6581-11-16 23497722 PMC3598176

[27] Jari M Qorbani M Moafi M Motlagh ME Keikha M Ardalan G. Association of 25-hydroxy Vitamin D levels with indexes of general and abdominal obesity in Iranian adolescents: The CASPIAN-III study. J Res Med Sci. (2015) 20:122. 25983762 PMC4400704

[28] Barchetta I De Bernardinis M Capoccia D Baroni MG Fontana M Fraioli A. Hypovitaminosis D is independently associated with metabolic syndrome in obese patients. PLoS ONE. (2013) 8:e068689. 10.1371/journal.pone.0068689 23935881 PMC3729690

[29] Kelishadi R Jamshidi F Qorbani M Motlagh ME Heshmat R Ardalan G. Association of hypertriglyceridemic waist phenotype with liver enzymes and cardiometabolic risk factors in adolescents: the CASPIAN-III study. J Pediatr. (2016) 92:512–20. 10.1016/j.jpedp.2016.06.014 27343636

[30] Miñambres I Sánchez-Hernández J Sánchez-Quesada JL Rodríguez J de Leiva A Pérez A. The association of hypovitaminosis D with the metabolic syndrome is independent of the degree of obesity. ISRN endocrinol. (2012) 2012:691803. 10.5402/2012/691803 23150833 PMC3485873

[31] Wortsman J Matsuoka LY Chen TC Lu Z Holick MF. Decreased bioavailability of vitamin D in obesity. Am J Clin Nutr. (2000) 72:690–3. 10.1093/ajcn/72.3.690 10966885

[32] Caron-Jobin M Morisset AS Tremblay A Huot C Légaré D Tchernof A. Elevated serum 25 (OH) D concentrations, vitamin D, and calcium intakes are associated with reduced adipocyte size in women. Obesity. (2011) 19:1335–41. 10.1038/oby.2011.90 21527900

[33] Ford ES Zhao G Tsai J Li C. Associations between concentrations of vitamin D and concentrations of insulin, glucose, and HbA1c among adolescents in the United States. Diabetes Care. (2011) 34:646–8. 10.2337/dc10-1754 21273498 PMC3041198

[34] Nunlee-Bland G Gambhir K Abrams C Abdul M Vahedi M Odonkor W. Vitamin D deficiency and insulin resistance in obese African-American adolescents. J Pediatr Endocrinol Metab. (2011) 24:29–33. 10.1515/jpem.2011.107 21528812 PMC5477057

[35] Vanga SR Good M Howard PA Vacek JL. Role of vitamin D in cardiovascular health. Am J Cardiol. (2010) 106:798–805. 10.1016/j.amjcard.2010.04.042 20816120

[36] Esteghamati A Aryan Z Nakhjavani M. Differences in vitamin D concentration between metabolically healthy and unhealthy obese adults: associations with inflammatory and cardiometabolic markers in 4391 subjects. Diabetes Metab. (2014) 40:347–55. 10.1016/j.diabet.2014.02.007 24811744

[37] Brenner DR Arora P Garcia-Bailo B Wolever TM Morrison H El-Sohemy D Plasma vitamin D levels and risk of metabolic syndrome in Canadians. Clin Investig Med. (2011) E377–84. 10.25011/cim.v34i6.15899 22129928

[38] Al-khalidi B Kimball SM Kuk JL Ardern CI. Metabolically healthy obesity, vitamin D, and all-cause and cardiometabolic mortality risk in NHANES III. Clin Nutr. (2019) 38:820–8. 10.1016/j.clnu.2018.02.025 29525513

[39] Moore CE Liu Y. Low serum 25-hydroxyvitamin D concentrations are associated with total adiposity of children in the United States: National Health and Examination Survey 2005 to 2006. Nutr Res. (2016) 36:72–9. 10.1016/j.nutres.2015.11.003 26773783

[40] Jorde R Grimnes G. Vitamin D and metabolic health with special reference to the effect of vitamin D on serum lipids. Prog Lipid Res. (2011) 50:303–12. 10.1016/j.plipres.2011.05.001 21640757

[41] Kelishadi R Farajzadegan Z Bahreynian M. Association between vitamin D status and lipid profile in children and adolescents: a systematic review and meta-analysis. Int J Food Sci Nutr. (2014) 65:404–10. 10.3109/09637486.2014.886186 24524677

[42] Saedisomeolia A Taheri E Djalali M Moghadam AM Qorbani M. Association between serum level of vitamin D and lipid profiles in type 2 diabetic patients in Iran. J Diab Metab Disord. (2014) 13:7. 10.1186/2251-6581-13-7 24398023 PMC3937161

[43] Heshmat R Hemati Z Payab M Hamzeh SS Motlagh ME Shafiee G. Prevalence of different metabolic phenotypes of obesity in Iranian children and adolescents: the CASPIAN V study. J Diabetes Metab Disord. (2018) 17:211. 10.1007/s40200-018-0363-5 30918857 PMC6405382

[44] Payab M Qorbani M Shahbal N Motlagh ME Hasani-Ranjbar S Zahedi H. Association of anthropometric indices with metabolic phenotypes of obesity in children and adolescents: the CASPIAN-V study. Front Endocrinol. (2019) 10:786. 10.3389/fendo.2019.00786 31849834 PMC6902658

[45] Payab M Hasani-Ranjbar S Merati Y Esteghamati A Qorbani M Hematabadi M. The prevalence of metabolic syndrome and different obesity phenotype in Iranian male military personnel. Am J Men's Health. (2017) 11:404–13. 10.1177/1557988316683120 28201955 PMC5675284

[46] Primeau V Coderre L Karelis A Brochu M Lavoie M Messier V. Characterizing the profile of obese patients who are metabolically healthy. Int J Obesity. (2011) 35:971. 10.1038/ijo.2010.216 20975726

[47] Wang B Zhuang R Luo X Yin L Pang C Feng T. Prevalence of metabolically healthy obese and metabolically obese but normal weight in adults worldwide: a meta-analysis. Hormone Metab Res. (2015) 47:839–45. 10.1055/s-0035-1559767 26340705

[48] Renzaho AM Halliday JA Nowson C. Vitamin D, obesity, and obesity-related chronic disease among ethnic minorities: a systematic review. Nutrition. (2011) 27:868–79. 10.1016/j.nut.2010.12.014 21704500

[49] Velho S Paccaud F Waeber G Vollenweider P Marques-Vidal P. Metabolically healthy obesity: different prevalences using different criteria. Eur J Clin Nutr. (2010) 64:1043. 10.1038/ejcn.2010.114 20628408

[50] Calori G Lattuada G Piemonti L Garancini MP Ragogna F Villa A. Prevalence, metabolic features, and prognosis of metabolically healthy obese Italian individuals: the Cremona Study. Diabetes Care. (2011) 34:210–5. 10.2337/dc10-0665 20937689 PMC3005463

[51] Wildman RP Kaplan R Manson JE Rajkovic A Connelly SA Mackey RH. Body size phenotypes and inflammation in the Women's Health Initiative Observational Study. Obesity. (2011) 19:1482–91. 10.1038/oby.2010.332 21233809 PMC3124587

[52] Chun S Lee S Son H-J Noh H-M Oh H-Y Jang HB. Clinical characteristics and metabolic health status of obese Korean children and adolescents. Korean J Fam Med. (2015) 36:233. 10.4082/kjfm.2015.36.5.233 26435814 PMC4591389

[53] Prince RL Kuk JL Ambler KA Dhaliwal J Ball GD. Predictors of metabolically healthy obesity in children. Diabetes Care. (2014) 37:1462–8. 10.2337/dc13-1697 24574347

